# Analysis of the Ocular Refractive State in Fighting Bulls: Astigmatism Prevalence

**DOI:** 10.1155/2017/8203269

**Published:** 2017-11-02

**Authors:** Juan M. Bueno, Matteo Lo Sapio, J. Manuel Sanes, Juan Seva

**Affiliations:** ^1^Laboratorio de Óptica, Instituto Universitario de Investigación en Óptica y Nanofísica, Universidad de Murcia, Campus de Espinardo, Ed. 34, 30100 Murcia, Spain; ^2^Anatomía y Anatomía Patológica Comparadas, Facultad de Veterinaria, Universidad de Murcia, Campus de Espinardo, 30100 Murcia, Spain

## Abstract

The purpose of this study was to describe the ocular refractive state (ORS) of fighting bulls. The study consisted of 90 ophthalmological healthy animals (85 in* post-mortem* and 5 in living conditions, resp.). The ORS of the eyes (2 per animal) was determined using streak retinoscopy.* In vivo* animals were assessed at a fighting bull farm facility.* Post-mortem* measurements were carried out at a local arena. The ORS along the horizontal meridian ranged between −1.00 and +2.50 diopters (D), with a mean of +0.66 ± 0.85 D in* post-mortem* animals. Values for* in vivo* conditions were similar (+0.75 ± 0.46 D). Left and right eyes were highly correlated in both sets (*p* < 0.001). A fairly good correlation was also observed when comparing living and* post-mortem* eyes in the same animals. Anisometropia ≥ 1.00 D was diagnosed in 3 animals. Astigmatism (≥+0.5 D) was detected in 93% of the eyes. To our knowledge, the ORS of the fighting bull has been reported for the first time. Although values vary among individuals, all eyes presented a marked astigmatism. Whereas the horizontal meridian was slightly hyperopic, the vertical meridian was always closer to emmetropia. These results represent a starting point to understand the ocular optics of this kind of animals, which might benefit the selection of animals at the farm before being sent to the bullfighting arena.

## 1. Introduction

Vision is one of the most important elements underlying the quality of life of humans and animals. In particular, the ocular refractive state (ORS) determines the proper functioning of the eye as an optical system [1]. The study of the ORS by optometry practitioners is a common practice in humans. Although ORS values vary greatly, the most common is myopia [2]. Animal models are not strictly comparable to humans, but the analysis of their ORS might provide additional information on the function and development of their visual system. Since the ocular globe size and the retinal structure differ between humans and animals, the same amount of ametropia could also provide different visual effects and have some impact on animal behavior. In animals, the ORS has often been measured with retinoscopy [3] or photorefraction [4].

The ORS of different species have been determined for a better understanding of the factors limiting visual performance. ORS studies ranged from the largest eyes in mammals such as the elephant [5], the rhinoceros [6], or the horse [7], to smaller ones (both domestic and nondomestic) such as dogs [8], cats [9], rats [10], rabbits [11], and monkeys [12] among others.

Moreover, the ORS in animals might be affected by different factors. Genetics might lead to particular refractive errors [13]. This has also been reported to depend on breed in canine, feline, and equine eyes [7–9]. Age also affects the ORS of dogs [8], cats [9], monkeys [14], and humans [15]. Moreover, the eyes of young mammals, birds, and lower vertebrate are also be influenced by the visual environment [16–18].

Although bovine eyes are often used for academic purposes, to our knowledge there is a lack of references regarding the ORS. Some authors reported data about visual acuity in cattle using psychophysical procedures and discrimination learning methods. Visual acuity was found to be poor (<0.1), but ORS values were not provided [19, 20].

A particular bovine breed such as fighting bull deserves a special attention. These animals are bred free range on extensive farms and under such experimental conditions require well-defined manipulation procedures with minimum human contact. Although they are genetically selected for a certain combination of physical energy and aggressiveness, their behavior usually differs significantly among animals (even brothers) during bullfighting [21]. Sometimes at the arena, the bull's behavior is considered to be “out of normal.” This is often attributed to an uncorrected (near or far) vision, what might be very dangerous for the “matador.” For many years, veterinarians, farmers, and bullfighters have been interested in this topic. In this sense, the knowledge of the fighting bull ORS would provide information on the ocular optics as the first step of the visual process and improve the selection of the most appropriate animals at the farm.

The purpose of the present study is to assess the ORS in a population of fighting bulls. The study demands few specific in vivo ocular measurements, which requires freedom to access the farm animals. To be more precise, the work herein includes a small set of living animals (measured at the farm) and a larger set to be measured in* post-mortem* conditions (immediately after bullfighting). Moreover, considering the special safety conditions and restricted entry to the bull farms, special legal commitments will be mandatory in order to instigate this study.

## 2. Materials and Methods

### 2.1. Animals and Procedure

**Figure 1:**
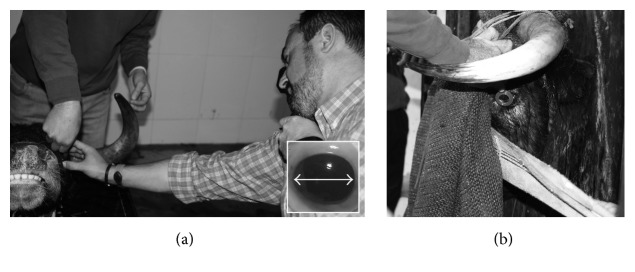
Streak retinoscopy carried out in* post-mortem* (a) and* in vivo* (b) conditions.

The experiment was divided into three parts. The first part of the experiment involved a total of 10 eyes from 5 living fighting bulls (group #1) obtained from a farm in the Region of Murcia, Spain. Ages ranged between 48 and 55 months. Animals with corneal edema or eyelid swelling were excluded from the selection. The ORS was measured using streak retinoscopy along the horizontal meridian (HM) (see inset in Figure 1(a)). This was carried out by a single investigator, using a hand held retinoscope (HEINE Optotechnik, Herrsching, Germany) and trial lenses. The eyes were examined at a working distance of 67 cm, and the results were therefore corrected by −1.50 D.  Figure 1(b) shows a photograph taken during measurements.

Due to security reasons, only veterinarians and bullfighters have admission to fighting bull farms in Spain. However, a special permission and a written consent gave us access to the farm for this experiment. Animals were tranquilized with an intramuscular injection of xylazine (10 mg/100 kg) before being boxed-in for periodical veterinary examination purposes. Then the ORS was determined. Due to xylazine's effects, the eyes were always under mydriasis [22]. This* in vivo* experiment was compared to the* post-mortem* measurements in the same animals (see below). This allowed corroborating the accuracy of the procedure. Both types of measurements (i.e.,* in vivo* and* post-mortem*) did not interfere with the regular activity of veterinarians at any time. The entire protocol was approved by both the Bullfighting Arena Veterinary Committee and the Department of Health (Region of Murcia) review board.

The ORS of these animals was also measured in* post-mortem* conditions at the local bullfighting arena in Murcia, Spain. Measurements were carried out at the arena slaughterhouse once the animal was removed from the bullring. Due to the* post-mortem* conditions the pupil was always under dilation. Eyes were not manipulated before or during examination. The time between death and assessment never exceeds 10 min.

A set of changes (both physiological and physical) occur after death. In particular, intraocular pressure varies [23], which might potentially affects the ocular globe and ORS measurements. A tonometer was used to measure the intraocular pressure in 10 eyes at 5, 10, and 20 minutes after death. No changes were detected during that time, which indicates that the influence of this parameter is negligible in the present experiment. Moreover,* post-mortem* conditions might also produce changes in retinal reflectivity and affect visualization of the retinal reflections used in retinoscopy. To test this, the ORS was also measured in 10 eyes at 5, 10, and 20 minutes after death. No changes were found.

The authors were not involved in the different steps of the fighting procedure at all. This type of sacrifice is authorised in Spain and France by the European Community legislation (Lisbon Treaty, art.13: […]* while respecting the legislative or administrative provisions and customs of Member States relating in particular to religious events, cultural traditions and regional heritage*).

In order to complete statistics on the ORS for* post-mortem* conditions, an additional set of animals (group #2) composed of 50 bulls was used. The ORS was measured along HM in the 100 eyes of these* post-mortem* animals at the local arena. The experimental procedure was the same as the one above described. Ages ranged from 43 to 70 months (mean: 54 ± 4), and weight between 480 and 572 kg. To assess the accuracy of the ORS results, 10 animals (20 eyes) were also examined by a second person. The ORS values obtained were the same for most of the eyes (when a difference existed, it was never larger than 0.25 D; interrater reliability 0.9).

Group #3 was used to test the presence of astigmatism in the eye of the fighting bull. For this experiment, the ORS was measured in 70 additional eyes from 35* post-mortem* animals along both the HM and the vertical meridians (VM) [24]. The conditions of measurements were the same as those used for group #2. Ages ranged from 40 to 60 months (mean: 53 ± 6), and weight between 412 and 568 kg. The animals of the three groups were male.

### 2.2. Data Analysis

ORS were expressed in diopters (D) as mean value ± standard deviation. A net refractive error ≥+0.50 D was considered as hyperopia; myopia was considered for an ORS of −0.50 D or less. When a difference of ≥1.00 D was measured between the two eyes of the same animal, anisometropia was reported. Astigmatism was defined as a difference higher than 0.5 D between the ORS values for HM and VM.

The Kolmogorov-Smirnov test was initially used to check the normal distribution of the data. Paired Student's *t*-test was performed to test the effects of the age and weight on the refractive state. This was also used to compare the ORS of both eyes. Linear regression was used to estimate the values that best fit a linear equation. Pearson's correlation coefficients were calculated to evaluate the degree of association between variables in the data set. Values of *p* < 0.05 were considered significant. Student's *t*-test for independent samples was performed to compare refractive state of groups #1 and #2. To compare the ORS along HM and VM, a Wilcoxon Rank-Sum test was applied.

## 3. Results

### 3.1. Refractive State along the Horizontal Meridian: Post-Mortem versus In Vivo

**Figure 2:**
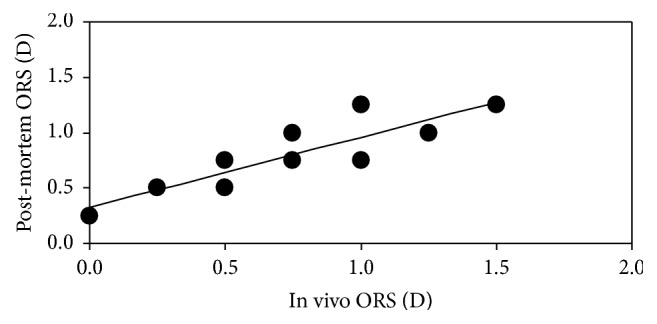
Comparison of ORS values between living and* post-mortem* conditions in the eyes of group #1. The line represents the best linear fit.

In the animals of group #1 (living bulls), the values of the ORS along HM ranged from 0.00 to +1.50, with an average of +0.75 D (standard deviation: ±0.46 D), which indicates the presence of hyperopia. These animals were also measured in* post-mortem* conditions after the regular fight. The comparison for living/*post-mortem* is shown in Figure 2. A significant linear correlation was found (*R* = 0.88, *p* < 0.001). This indicates that, despite the physiological changes produced when the animal dies, there is a fairly good agreement between the ORS assessed before and after death.

**Figure 3:**
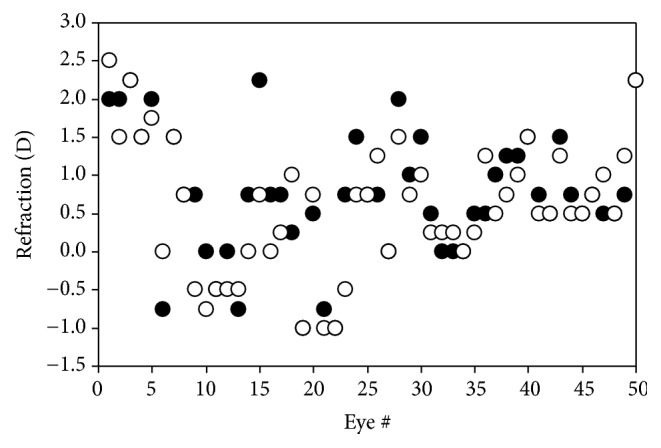
ORS measured along HM for the eyes of group #2 (*N* = 100,* post-mortem* conditions). Black and white symbols correspond to REs and LEs, respectively.

**Figure 4:**
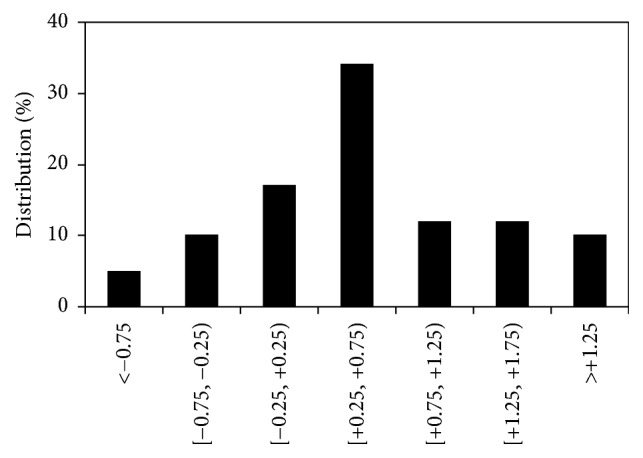
Distribution of spherical refraction in the eyes of group #2 (*N* = 100). Square brackets by a value indicate that the actual value is included within the corresponding interval. On the contrary, round brackets indicate that the value is not included.

Figure 3 depicts the values of net spherical refraction for all eyes of group #2. Right (RE) and left eyes (LE) are labelled in black and white, respectively. Values ranged from −1.00 to +2.50 D, with a mean of +0.66 D (standard deviation: ±0.85 D). 15 eyes (15%) were myopic (i.e., refraction ≤ −0.5 D). Mean values for REs and LEs were, respectively, +0.74 ± 0.85 D and +0.59 ± 0.84 D. There are no statistically significant differences between both eyes (*p* = 0.31). Moreover, the ORS values did not present any correlation with age (*p* = 0.46) or weight (*p* = 0.23). For completeness, Figure 4 shows the distribution of the refractive errors across all eyes of this group #2: 68% of the eyes presented an ORS in the hyperopic range (ORS ≥ +0.50 D). This is similar to the result obtained in group #1, where the measured eyes tended to be also slightly hyperopic.

**Figure 5:**
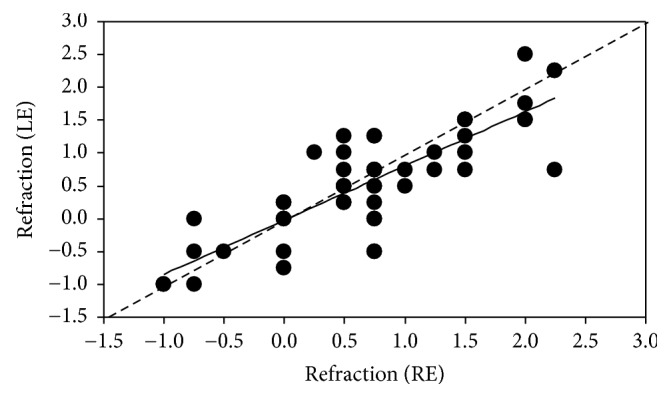
Comparison of the refractive error between RE and LE for the fighting bulls of group #1. The line represents the best linear fit (*R*_LE_ = 0.84*∗R*_RE_ + 0.02). Dashed line is 1 : 1.

In order to analyze a possible symmetry between both eyes, Figure 5 presents the values of refraction along HM in REs plotted against the magnitude in LEs. There was a significant linear correlation between the spherical refraction values of REs and LEs (*R* = 0.83, *p* < 0.001). Anisometropia (difference in refractive error for a pair of eyes ≥ 1 D) was diagnosed in 3 animals with a maximum value of 1.50 D.

**Figure 6:**
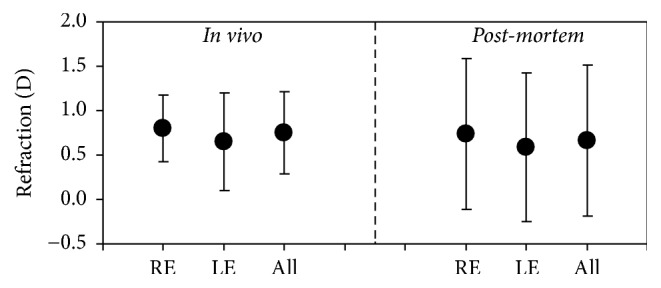
Comparison of averaged ORS values between* in vivo* (group #1) and* post-mortem* (group #2) eyes. Error bars represent the standard deviation.

For the sense of completeness, Figure 6 presents the averaged ORS values for all animals of groups #1 and #2. No significant differences were found between both sets of eyes (*p* = 0.31).

### 3.2. Refractive State along Two Perpendicular Meridians: Astigmatism

**Figure 7:**
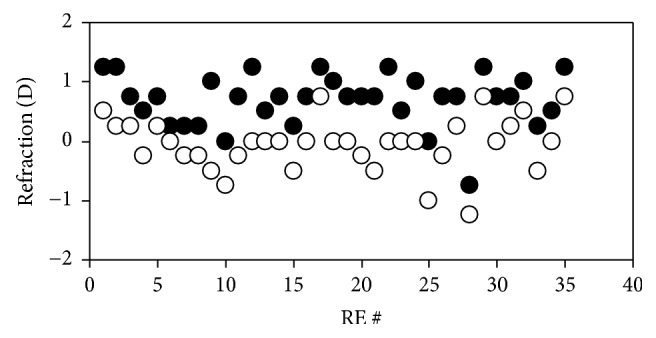
ORS values measured in* post-mortem* conditions along HM (black symbols) and VM (white symbols) for all the REs of animals of group #3.

**Figure 8:**
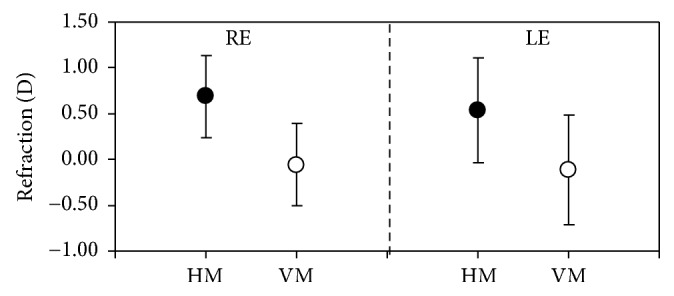
Averaged ORS values for both meridians across all eyes of group #3. Error bars represent the standard deviations.

The ORS values for the REs of animals in group #3 measured along both meridians, HM and VM, are depicted in Figure 7. For this group the ORS values along HM varied between −0.75 and +1.25 D, with a mean of +0.69 ± 0.45 D, an average value comparable to that obtained in group #2 (see Table 1 for a direct comparison). From the plot, it can also be observed that, for all these eyes, the VM was always “less hyperopic” than the HM (average for VM: −0.06 ± 0.45 D). A similar behavior was found for LEs. For a better comparison, Figure 8 shows the mean ORS for both eyes and meridian across all animals of group #3. Differences between HM and HV were significantly different for both RE and LEs (*p* < 0.001, Wilcoxon Rank-Sum test).

**Figure 9:**
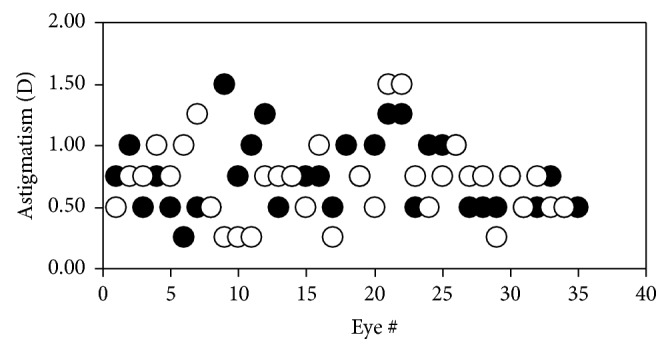
Values of astigmatism for the eyes of group #3. Black and white symbols correspond to REs and LEs, respectively.

Figure 9 plots the difference in ORS measured along HM and VM for all eyes. Mean values were +0.75  ±  0.29 and +0.70  ±  0.32 D for RE and LE, respectively. Astigmatism ≥ 0.50 D was present in 65 of 70 eyes (93%).

**Figure 10:**
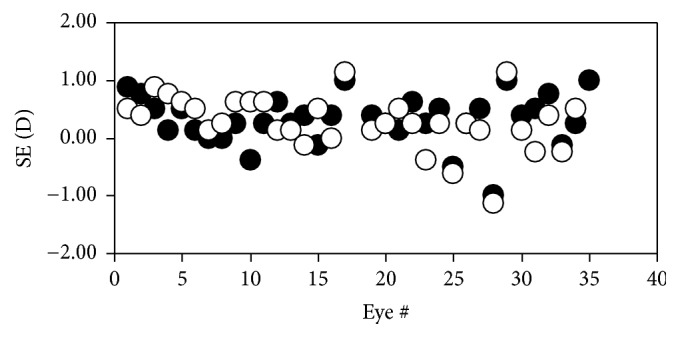
Values of SE for all eyes of group #3. The labels are the same as in the previous figure.

The spherical equivalent (SE) of an ocular refraction is equal to half of the algebraic sum of the ORS values along two perpendicular meridians. The image corresponding to this SE is the circle of least confusion, which can be understood as the “best image” provided by an astigmatic optical system. The actual SE values are depicted in Figure 10. The mean value was +0.31 ± 0.43 D for REs and +0.27 ± 0.46 D for LEs.

## 4. Discussion and Conclusions

A retinoscope was used to measure the ORS in fighting bulls. To our knowledge, no studies have been conducted to examine the ORS in bovine eyes (fighting bulls in particular) and results here reported cannot be compared with previous literature.

The ORS was measured in both* in vivo* and* post-mortem*. The reasons for the difference in the number of animals can easily be understood. Spanish Bullfighting Regulation is very restrictive in terms of animal management and human access to the farm facilities. Fighting bulls are very dangerous animals that grow in large outdoor fenced extensions. Getting permissions for a scientific work with living fighting bulls is challenging, with long and complex procedures. At the farm, the bulls involved in the present measurements were not isolated for the experiment itself. The measurements were carried out in a veterinary control (see Section 2 above). The permission given to the authors was very restrictive. The protocol was very limited in terms of maximum time allowed to measure each animal. Due to this, the ORS values were exclusively measured along the HM in living animals. However, for the present work, the most important point is that these* in vivo* measurements can be used to validate the results obtained in* post-mortem* conditions (Figures 2 and 6).

Previous literature also reports ORS values in small sets of animals, such as 6 elephants [5], 4 rhinoceros [6], or 3 turtles [25].

Our results on ORS (along HM) show a slight tendency towards hyperopia with mean values of +0.66 ± 0.85 and +0.75 ± 0.46 D in* post-mortem* and* in vivo* conditions, respectively. These similar values validate the* post-mortem* results, indicating that they are reliable and consistent. The refraction of 68% of* post-mortem* and 8 out of 10 living eyes was ≥+0.50 D. The smaller variability among* in vivo* eyes (see Figure 6) might be attributed to the reduced size of the sample under study.

Previous literature has reported ORS measurements in a wide number of animals. Domestic animals such as cats and dogs have been reported to be myopic (–0.78 ± 1.37 D) [9] and emmetropic (–0.05 ± 1.36 D) [8], respectively. In dogs, the ORS was shown to vary with breed. Breeds used in activities requiring highly visual functioning were found to be closer to emmetropia.

The ORS of big mammals such as the elephant [5] and the horse was close to emmetropia (+0.23 and −0.17 D, resp.) [7]. No difference was observed between cyclopleged and noncyclopleged eyes in these animals. The rhinoceros was found to be mildly hyperopic (from +0.75 to +1.5 D) [6]. ORS values in small mammals such as rats were clearly hyperopic [10].

Apart from factors such as genetics or breed (race in humans), age might have an important influence on the ORS in both humans and animals [8, 9, 14, 15]. No relationship between age and ORS was found in the present work. This might be due to the reduced age range of the samples. No data on bovine ocular growth have been found by these authors. Although fighting bulls older than 4 years are considered as adults, we ignore if the eyes here measured had reached a stable size. At this point, as a next step, it would be interesting to measure both the ORS and the ocular dimensions as a function of age in fighting bulls. This would inform on the evolution of the bull's ocular globe and changes of the refractive errors with age might also be justified.

An unbalance of the ORS between pairs of eyes might lead to problems with the development of binocularity. This has been detected in humans [26], horses [7], and monkeys [12], among others. Although different authors have reported anisometropia in dogs [8, 27], they did not agree in the prevalence, which ranged between 6% and 25%. In the animals here screened, only 3 presented anisometropia. This suggests that the prevalence of anisometropia is low among fighting bulls. To the best of our knowledge, the effect of anisometropia on binocular vision and stereoacuity has not been described in bovines to date.

Previous literature has reported astigmatism in humans [28] and elephants [5]. Dogs also presented astigmatism [27], although the prevalence depended on the breed [8].

Since a complete ocular refraction exam requires the assessment of two perpendicular meridians, the presence of astigmatism was analyzed in group #3. Results here reported show that astigmatism is an important contributor to image quality in the bull eye. In particular, 93% of the eyes presented astigmatism (≥0.50 D). The values ranged between +0.25 and +1.5 D (Figure 9), with a mean of +0.73 ± 0.30 D.

It is interesting to notice that the ORS along VM was found to be close to emmetropia on average (Figures 7 and 8). Since no previous data have been found, the reason for this is unknown. However, this might be related to the fact that the bovine retina presents a visual horizontal streak where most of the retinal photoreceptors are located [29]. Since the bull's eye is an astigmatic optical system, the image provided by the VM will be oriented along the horizontal direction and will be placed near the retinal plane (since HM is mainly hyperopic, the image corresponding to the HM will be located behind the retina). Although further experiments are required, it is possible that the slit-shaped pupil of bovine is also related to both the retinal horizontal streak and the astigmatic nature of the ocular optics. A similar funding was reported in turtles: refraction values along VM were more myopic than those along HM [25]. The authors claimed that the reason for astigmatism was unknown, although they referred to anatomical investigations of the retina and the presence of a horizontal stripe of increased ganglion cell density.

However, the most appropriate way to understand the optical behavior of an astigmatic system is to analyze the location of the circle of least confusion. As we claimed above, in optometric terms, the optical power of this circle is known as the SE and corresponds to the “sharpest image” provided by an astigmatic system. Although the SE value is particular for each eye, the averaged value for all eyes was +0.29 ± 0.44 D. It has been reported that bovine eyes have little accommodative ability, ~2 D [30]. Then, it could be believed that the animal can accommodate and compensate for this remaining ~1/4 D in order to focus fairly well objects located at far distances.

The analysis of the accommodative response is out of the scope of this work and these authors do not have enough data to corroborate the existence of 2 D of accommodation amplitude in the bull eye. If this is the case, the closest distance where the ocular optics gives “sharp” images on the retina would be about 60 cm. It is necessary to point out that this distance must be understood in a general sense, since each eye presents its own ORS. However this fact gives an idea about the interval of vision of the fighting bull. Furthermore, additional experiments on the optical properties of the crystalline lens would allow investigating if the contribution of its spherical aberration increases the depth of focus of the bull's eye. This would benefit the vision of objects located at different distances when reduced (or a lack of) accommodation capabilities are present [31].

During the regular fighting, bullfighters and attendees often complain to the arena's President about the bulls' visual performance (Spanish Regulation allows for this, but only the President can take the final decision of changing the animal by another one). The reasons for this are mainly based on the fact that the animal shows noticeable uncorrected (far or near) vision. This leads to an unpredictable behavior which might put the bullfighter at very high risk.

It is obvious that the reported results on ORS themselves cannot justify the behavior of the bull in the arena. However, if the Spanish Bullfighting Regulation is modified in the next future, measurements of the ORS should be included in the regular veterinarian* in vivo* examinations at the farm, before the animals are brought to the arena facilities. Extra expenses would be probably avoided (some thousands of euros in total) which are generated when the bull (once in the bullring) has to be returned due to visual problems affecting the usual fighting and the bull being extremely dangerous for the bullfighter.

To conclude, the ORS of the adult fighting bull has been measured in* in vivo* and* post-mortem* conditions for the first time. Results show the presence of astigmatism, with a mild hyperopia along HM and a VM closer to emmetropia. Although these measurements represent a first step in understanding the ocular optics of the fighting bull, it is worth noticing that a reduced set of living biological specimens cannot provide definitive conclusions. The nature of these animals has been a limitation for the results reported here. However, further studies with more living animals will lead to more definitive conclusions. Since streak retinoscopy is a time-consuming technique for this kind of animals, alternative objective techniques such infrared photorefraction might be more appropriate [4, 24] in future experiments.

## Figures and Tables

**Table 1:** Summary of the ORS values for the three groups of animals involved in the study.

	ORS_#1_ (HM)	ORS_#2_* (in vivo)*	ORS_#3_ (HM)	ORS_#3_ (VM)
Max	+2.50	+1.50	+1.25	+1.25
Min	−1.00	0.00	−0.75	−1.50
Mean (±SD)	+0.66 ± 0.85	+0.75 ± 0.46	+0.65 ± 0.44	−0.06 ± 0.50
Mean LE (±SD)	+0.59 ± 0.84	+0.65 ± 0.55	+0.61 ± 0.45	−0.07 ± 0.55
Mean RE (±SD)	+0.74 ± 0.85	+0.85 ± 0.38	+0.69 ± 0.45	−0.06 ± 0.45
